# Headache associated with COVID-19: Frequency, characteristics and association with anosmia and ageusia

**DOI:** 10.1177/0333102420966770

**Published:** 2020-11-04

**Authors:** Pedro Augusto Sampaio Rocha-Filho, João Eudes Magalhães

**Affiliations:** 1 Hospital Universitario Oswaldo Cruz, Universidade de Pernambuco (UPE), Recife, Brazil; Division of Neuropsychiatry, Centro de Ciências Médicas, Universidade Federal de Pernambuco (UFPE), Recife, Brazil; 2 Hospital Universitario Oswaldo Cruz, Universidade de Pernambuco (UPE), Recife, Brazil; Faculdade de Ciências Médicas, Universidade de Pernambuco (UPE), Recife, Brazil

**Keywords:** Headache, COVID-19, SARS-CoV-2, olfaction disorders, anosmia, ageusia

## Abstract

**Objectives:**

To assess the frequency and characteristics of headache in patients with COVID-19 and whether there is an association between headache and anosmia and ageusia.

**Methods:**

This was a cross-sectional study. Consecutive patients admitted to hospital with COVID-19, confirmed by reverse transcription polymerase chain reaction (RT-PCR) technique, were assessed by neurologists.

**Results:**

Seventy-three patients were included in the study, 63% were male; the median age was 58 years (IQR: 47–66). Forty-seven patients (64.4%) reported headaches, which had most frequently begun on the first day of symptoms, were bilateral (94%), presenting severe intensity (53%) and a migraine phenotype (51%). Twelve patients (16.4%) presented with headache triggered by coughing. Eleven (15%) patients reported a continuous headache. Twenty-eight patients (38.4%) presented with anosmia and 29 (39.7%) with ageusia. Patients who reported hyposmia/anosmia and/or hypogeusia/ageusia experienced headache more frequently than those without these symptoms (OR: 5.39; 95% CI:1.66–17.45; logistic regression). Patients with anosmia and ageusia presented headache associated with phonophobia more often compared to those with headache without these complaints (Chi-square test; *p* < 0.05). Headache associated with COVID-19 presented a migraine phenotype more frequently in those experiencing previous migraine (*p* < 0.05).

**Conclusion:**

Headaches associated with COVID-19 are frequent, are generally severe, diffuse, present a migraine phenotype and are associated with anosmia and ageusia.

## Introduction

Although the symptoms of COVID-19 are predominantly respiratory, symptoms and complications in the central and peripheral nervous system have increasingly been described, including anosmia, ageusia and headache (1). These complications are possibly caused by direct viral injury, immunological mechanisms and by hypoxia (2).

It is estimated that with the COVID-19 pandemic there has been a five-fold increase in the incidence of headache in the affected regions (3). In most studies, the prevalence of headache in patients with COVID-19 is around 12% (4–8). Little is known about the characteristics of these headaches. An Italian study designed to assess neurological symptoms in hospitalized patients reported a more frequent pattern similar to a tension-type headache (9). These headaches are often bilateral (10). In our clinical practice, we have observed a higher frequency of headache than reported in the literature, which is often one of the most disabling symptoms.

One of the potential routes for SARS-Cov-2 to enter the central nervous system is through invasion of cranial nerves. This occurs mainly through the olfactory pathway (11). Olfactory dysfunction occurs in 52.7% (95% CI: 29.6–75.2%) of patients with COVID-19 and gustatory dysfunction in 43.9% (95% CI: 20.5–68.9%) (12).

Magnetic resonance studies on patients with COVID-19 and anosmia have reported changes in the olfactory bulb and in structures linked to olfaction (13–15). In one study, four of the five patients included underwent magnetic resonance imaging due to persistent headache (15). Recently, we described a case of a patient with COVID-19 who developed a new daily persistent headache. Anosmia was associated with facial pain and the headache began one day after anosmia, which persisted for more than 3 months (16).

This study aims to assess the frequency and characteristics of headache presented by patients hospitalized for COVID-19 and whether there was an association between headache and complaints of anosmia and ageusia.

The hypotheses of this study are that patients with COVID-19 have a high frequency of headache, that patients with olfactory and gustatory dysfunction present a higher frequency of headache and that these headaches have different characteristics when compared with those who do not present with olfactory or gustatory complaints.

## Methods

### Study type

This was a cross-sectional study.

### Eligibility criteria

Patients admitted to the Hospital Universitário Oswaldo Cruz diagnosed with COVID-19 confirmed by reverse transcription polymerase chain reaction (RT-PCR) technique from material collected by nasal and oropharynx swab were included in the study. Patients were tested with different RT-PCR kits, according to the availability of the public health system at the time of the assessment: Molecular Kit SARS-Cov-2(E/P1) (Bio-Manguinhos, Fiocruz, Rio de Janeiro, Brazil), BIOMOL Kit OneStep/COVID-19 (IBMP, Paraná, Brazil), AllplexTM 2019-nCov Assay (Seegene Inc., Minas Gerais, Brazil), and 2019-nCov CDC Assay (IDT Inc., Iowa, USA), respectively with a detection limit of 50, 6, 100, and 8 copies per reaction. The tested specificity of all kits was > 99%.

Patients were excluded if they presented a decreased level of consciousness, confusional state, and those with cognitive impairment that impeded the reporting of symptoms or diagnosed with other secondary headaches.

### Location

Context: The Hospital Universitário Oswaldo Cruz is a university hospital linked to the Universidade de Pernambuco and is a reference center for infectious diseases in the state of Pernambuco, Brazil. During the COVID-19 pandemic, all doctors on the hospital clinical staff, regardless of their specialties, were called upon to attend patients with COVID-19 admitted to this hospital. Thus, neurologists were also brought in, and were responsible for monitoring these patients who were admitted to the hospital wards.

### Data collection

Patients were assessed between 5 May and 17 June 2020 by two neurologists with experience in diagnosing and treating headaches. Interviews were performed after the first week of admission when the patients were usually stable, and RT-PCR results were available. For data collection, a semi-structured questionnaire was used that contained sociodemographic data, information regarding the duration of the COVID-19 symptoms and on the occurrence and characteristics of headache presented during COVID-19; and on the presence, manner of onset and evolution of anosmia and ageusia. Patients who presented with headaches associated with COVID-19 were questioned on the presence and characteristics of previous headaches. Patients reporting previous headaches were asked whether they considered the headache associated with COVID-19 to be similar or different to previous headaches.

During the physical examination, in order to avoid increasing the risk of contaminating the evaluator, ophthalmoscopy and olfactory and taste testing were not performed.

### Classification of headache disorders

Previous headaches presented by these patients were classified according to the third edition of the International Classification of Headache Disorders (ICHD-3) (17).

Headaches were classified as being associated with COVID-19 if they fulfilled criteria A to C1 for ‘Headache attributed to systemic viral infection' (9.2.2) (17).

The phenotypes of headaches associated with COVID-19 headache were classified as follows:

- Migraine phenotype: Headaches that fulfilled criteria B to D for migraine without aura (1.1). Headaches that did not meet criterion B because they lasted more than 72 hours were also considered.
- Tension-type headache phenotype: Headaches that fulfilled criteria B to D for tension-type headache (2.1). Headaches that did not meet criterion B because they lasted more than 7 days were also considered.
- Cough headache phenotype: Headaches that fulfilled criteria B to D for cough headache (4.1). Those headaches that were aggravated only after coughing were not classified as cough headache (17).

### Ethical considerations

All patients gave their informed consent and the study was approved by the Research Ethics Committee of Hospital Oswaldo Cruz (CAAE: 31071020.9.0000.5192; approval number: 4.004.564). Anonymized data will be shared on request from any qualified investigator.

### Statistical analysis

The statistical analyses were performed using SPSS Statistics Software version 21.0 (IBM Corporation, Armonk, NY, USA).

Quantitative data were tested regarding normality of distribution, by means of the Kolmogorov-Smirnov test. When the distribution was normal, the means and standard deviations were calculated. If not, the medians and the 25th and 75th percentiles were used (P_25_; P_75_).

The percentage distribution of the categorical variables was compared between the groups by means of the Chi-square test or Fisher’s exact test. Numerical variables were compared using the Mann-Whitney test. All tests were levelled using a 0.05 significance.

Logistic regression models were used to assess whether anosmia and ageusia were associated with headache. For these analyses, age and sex were also included as explanatory variables. Multiple testing in the final regression models was corrected using the analyses of 1000 computer-generated bootstrap samples.

## Results

**Figure 1.**
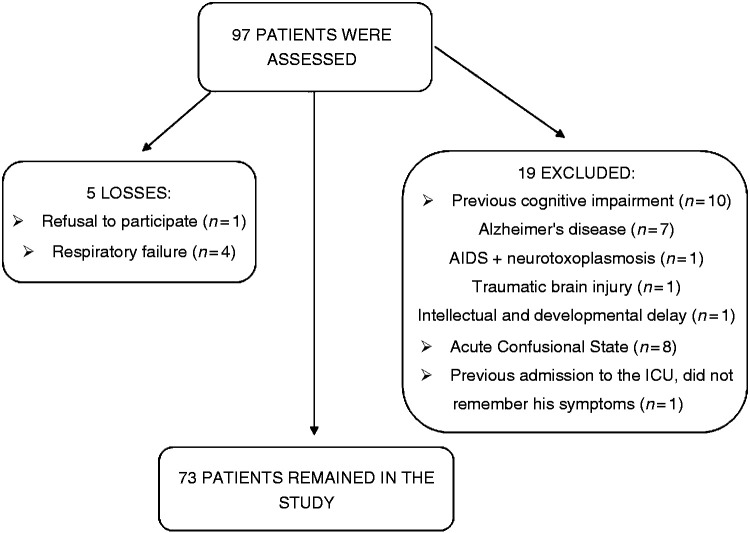
Flow chart of the study.

Ninety-seven patients were assessed, all of whom presented with respiratory symptoms and had tested RT-PCR positive for SARS-CoV-2 by nasal and oropharyngeal swab. The reasons for losses and exclusions are presented in Figure 1. There were five losses and 19 patients were excluded.

Seventy-three patients were included in the analysis. None of the patients presented with impaired consciousness, meningeal or focal neurologic signs. No patient underwent brain computed tomography, magnetic resonance imaging or cerebrospinal fluid analysis because they presented no warning signs for neurological complications of COVID-19.

These patients presented symptoms of COVID-19 for 15 days (IQR: 10.5–21) and had a median age of 58 years (IQR: 47–66); 46 (63%) were male.

### Headaches

Forty-seven patients (64.4%; 95% CI: 52.3–75.3) presented with headache associated with COVID-19. Table 1 presents the characteristics of these headaches. Most patients reported a headache on the first day of symptoms (range 1–20 days), very intense bilateral pain with a migraine phenotype.

**Table 1.** Characteristics of headaches associated with COVID-19.

Characteristics	(n = 47)
Headache onset (days)*, median (IQR)	1 (1.5–2)
Without previous headache	17 (36%)
Headache location**	
Frontal	38 (80%)
Temporal	26 (55%)
Parietal	23 (49%)
Occipital	17 (36%)
Bilateral headache	44 (94%)
Headache intensity	
Mild	6 (13%)
Moderate	16 (34%)
Severe	25 (53%)
Characteristic of headache	
Pulsatile quality	24 (51%)
Pressing/tightening quality	20 (43%)
Stabbing quality	2 (4%)
Do not know	1 (2%)
Worsening with physical activity	25 (53%)
Nausea	15 (32%)
Vomiting	5 (11%)
Photophobia	21 (45%)
Phonophobia	14 (30%)
Headache phenotype***	
Migraine-like	24 (51%)
Tension-type headache-like	19 (40%)
Cough headache	12 (26%)

*Counting from the first symptom.

**Patients could report more than one answer.

*** Cough headache patients may also have had another headache pattern.

Twelve of the 73 patients (16.4%; 95% CI: 8.8–27%) presented headache triggered by coughing. Of these, four only had cough headache, four also had a migraine phenotype, and four had a tension-type headache phenotype.

Eleven of the 73 patients (15%; 95% CI: 7.8–25.4%) presented a continuous headache (six with severe pain, four with moderate pain and one with mild pain), with a median period of 15 (IQR: 5–20) days of pain.

Among those in which the headache was not continuous, the median duration of headache attacks was 120 minutes (IQR: 90–1080).

Of the 47 patients, 30 (64%) reported headache prior to COVID-19 (18 experienced a tension-type headache and 12 migraine). Twenty-four (80%) of these patients rated the current headache as being different from previous headaches (self-evaluation). Of the 12 who had a previous migraine, 10 (83%) had headache associated with COVID-19 with a phenotype similar to migraine. Of the 18 who had a previous tension-type headache, nine (50%) had headache associated with COVID-19 with a phenotype similar to a tension-type headache.

Patients with headache were significantly younger (median: 56; IQR: 44–65.5 vs. 62; IQR: 52–74.5; Mann-Whitney test; *p* = 0.039). There was no difference regarding sex (male: 28/47: 59.6% vs. 18/26: 69.2%; Chi-square test; *p* = 0.413) or duration of symptoms (median: 14 days; IQR: 10–19.5 vs. 18.5; IQR: 12–25.5; Mann-Whitney test; *p* = 0.072) compared with patients who did not present with headache.

Twelve patients were still experiencing headache at the time of assessment, with a median duration of 10.5 days (IQR: 5–18). Of these patients, eight stated that the headache had been improving, two that it was unchanged and two that it was becoming worse. Patients who no longer had headache had reported pain for a median of 5 days (IQR: 4–7).

### Hyposmia/anosmia

Twenty-eight patients (38.4%; 95% CI: 27.2–50.5) reported anosmia (n = 24) or hyposmia, which in 15 (54%) patients began suddenly. Ten patients still reported anosmia/hyposmia at the time of the assessment, which had already lasted a median of ten (IQR 6–17.5) days. Those who no longer reported anosmia/hyposmia spent a median of five (IQR: 3.5–8) days with anosmia/hyposmia.

There was no difference regarding gender (male: 19/28: 68% vs. 27/45: 60%; Chi-square test; *p* = 0.499), age (median: 56.5; IQR: 46–61 vs. 61; IQR: 49.5–72.5; Mann-Whitney test; *p* = 0.052) or duration of symptoms until the time of assessment (median: 13.5 days; IQR: 8–19 vs. 17; IQR: 12.5–23; Mann-Whitney test; *p* = 0.178) when compared to those who reported no anosmia/hyposmia.

### Hypogeusia/ageusia

Twenty-nine patients (39.7%; 95% CI: 28.5–51.9) reported hypogeusia or ageusia (n = 19), 22 (76%) also had anosmia. Those who no longer presented hypogeusia/ageusia had spent a median of five (IQR: 4.5–8.5) days with hypogeusia/ageusia. Nine (31%) patients still reported the complaint at assessment, which had already lasted a median of 10 (IQR 6–14) days.

There was no difference regarding gender (men: 20/29: 69% vs. 26/44: 59%; Chi-square test; *p* = 0.499), age (median: 57; IQR: 54–64.5 vs. 60; IQR: 43–71; Mann-Whitney test; *p* = 0.463) or duration of symptoms until the time of assessment (median: 14 days; IQR: 10.5–20.5 vs. 16; IQR: 11–22; Mann-Whitney test; *p* = 0.711) when patients were compared with those who reported no hypogeusia/ageusia.

### Relationship between headache and the presence of changes in smell, taste, and previous migraine

Twenty-four patients (33%) presented with headache and hyposmia/anosmia. Sixteen (67%) of these reported that the two complaints had started on the same day. Patients with hyposmia/anosmia more often presented headache when compared to those who reported no hyposmia/anosmia (headache: 24/28: 86% vs. 23/45: 51%; OR: 5.7, 95% CI: 1.7–19.2; Chi-square test; *p* = 0.003).

Twenty-five patients (34%) presented with headache and hypogeusia/ageusia. Moreover, patients with hypogeusia/ageusia presented with headache more frequently than those who did not (headache: 25/29: 86% vs. 22/44: 50%; OR: 6.3, 95% CI: 1.9–20; Chi-square test; *p* = 0.002).

Patients who reported hyposmia/anosmia and/or hypogeusia/ageusia experienced headache more frequently than those without these symptoms (headache: 29/35: 83% vs. 18/38: 47%; OR: 5.4, 95% CI: 1.8–15.9; Chi-square test; *p* = 0.002). After controlling for confounding variables, hyposmia/anosmia and/or hypogeusia/ageusia were found to be statistically significantly associated with headaches (Table 2).

**Table 2.** Multivariate analysis for association between headache and anosmia and/or ageusia.

	Unadjusted odds ratio	*p*-value	Adjusted odds ratio	*p*-value
Female	1.53 (0.55–4.21)	0.413	–	
Age (<60 years)	2.82 (1.71–19.23)	0.037	–	
Hyposmia/anosmia	5.73 (0.11–13.72)	0.002	4.93 (1.37–17.8)*	0.015
Hypogeusia/ageusia	6.25 (1.86–20.95)	0.002	6.09 (1.73–21.45**	0.005
Hyposmia-anosmia/hypogeusia-ageusia	5.37 (1.81–15.9)	0.002	5.39 (1.66–17.45)***	0.005

*Model 1: Adjusted for anosmia, age, and sex.

**Model 2: adjusted for ageusia, age, and sex.

***Model 3: adjusted for anosmia/ageusia, age, and sex.

No association was observed between having a previous migraine and having had hyposmia/anosmia (previous migraine: 8/12: 67% vs. 16/35: 46%; Chi-square test; *p* = 0.210) or hypogeusia/ageusia (previous migraine: 8/12: 67% vs. 17/35: 49%; Chi-square test; *p* = 0.278) among those with headache associated with COVID-19.

Table 3 assesses the association between hyposmia/anosmia, hypogeusia/ageusia, and previous migraine with the characteristics of headache associated with COVID-19. Patients with hyposmia/anosmia and hypogeusia/ageusia presented significantly more phonophobia when compared to those who did not report these complaints. Patients with previous migraines reported that their headaches associated with COVID-19 became significantly worse with physical activity, nausea, and more had a migraine phenotype of headache associated with COVID-19 than those with no previous migraines.

**Table 3.** Comparison of headache characteristics according to Hyposmia/anosmia, Hypogeusia/ageusia and previous migraine.

Characteristics	Hyposmia/ anosmia		*p*-value	Hypogeusia/ageusia			Previous migraine		
	Yes (n = 24)	No (n = 23)		Yes (n = 25)	No (n = 22)	*p*-value	Yes (n = 12)	No (n = 35)	*p*-value
Headache onset (days), median (IQR)	1 (1.5–2)	1 (1.5–4)	0.394	1 (1–2)	1 (1–4)	0.192	1 (1–4)	1 (1–2.5)	0.921
Bilateral headache	24 (100%)	20 (87%)	0.067	24 (96%)	20 (91%)	0.476	11 (92%)	33 (94%)	0.749
Continous headache	6 (25%)	5 (22%)	0.792	4 (16%)	7 (32%)	0.201	5 (42%)	6 (17%)	0.083
Headache intensity									
Mild/moderate	10 (42%)	12 (52%)	0.471	11 (44%)	11 (50%)	0.681	3 (25%)	19 (54%)	0.079
Severe	14 (58%)	11 (48%)		14 (56%)	11 (50%)		9 (75%)	16 (46%)	
Worsening with physical activity	15 (63%)	10 (44%)	0.192	15 (60%)	10 (46%)	0.319	10 (83%)	15 (43%)	0.015
Nausea	8 (33%)	7 (30%)	0.831	10 (40%)	5 (23%)	0.205	7 (58%)	8 (23%)	0.023
Vomiting	4 (17%)	1 (4%)	0.171	4 (16%)	1 (5%)	0.204	2 (17%)	3 (9%)	0.433
Photophobia	14 (42%)	7 (30%)	0.055	14 (56%)	7 (32%)	0.096	8 (67%)	13 (37%)	0.076
Phonophobia	11 (46%)	3 (13%)	0.014	11 (44%)	3 (14%)	0.023	6 (50%)	8 (23%)	0.076
Headache pattern									
Migraine-like	14 (61%)	10 (50%)	0.474	15 (63%)	9 (47%)	0.321	10 (83%)	14 (45%)	0.024
Tension-type headache-like	9 (39%)	10 (50%)		9 (38%)	10 (53%)		2 (17%)	17 (55%)	

## Discussion

Sixty-four percent of our patients presented with headache, which generally began at the onset of symptoms, was bilateral, of moderate or severe intensity, throbbing and with a migraine phenotype. It should be mentioned that 15% of the patients presented a continuous headache, which was generally moderate to severe and lasted for at least 15 days. These data demonstrate that headache is not a “minor symptom”, and is a significant complaint that deserves to be actively investigated and treated.

The frequency of headache observed in the present study was much higher than that reported in most studies (4–8). Since most of these studies have assessed symptoms of COVID-19 in general, it is possible that the frequency of headache is underestimated because of respiratory symptoms, for which patients are most frequently admitted to hospital.

The prevalence of headache was calculated at 10.9% (8.6–13.5%) in a meta-analysis of 6486 patients included in 21 studies, in which the prevalence ranged from 3.5–34% (18). Three studies that assessed neurological symptoms in patients admitted with COVID-19 reported higher frequencies of headache (27% (10), 39% (19) and 43% (9)). A retrospective cohort study that assessed the prognostic value of headache concerning mortality found a headache frequency of 24% (20). One European multicenter study that assessed symptoms of patients hospitalized with COVID-19 reported a frequency of headache similar to that of the present study (70%) (21).

All patients were treated with ceftriaxone, azithromycin, and oseltamivir. Although headache is a rare adverse effect of these drugs, we cannot rule out that some patients may have had headaches for this reason. However, the headaches in 90% of patients started before hospitalization.

Migraine-like headaches have already been described in association with viral infections (22–26). For our patients who experienced previous migraines, the headache they experienced associated with COVID-19 was most often of a migraine phenotype. Migraine may be considered as an inherited disorder that involves alterations in sensory processing (27). Although cortical spreading depression has not yet been described as a consequence of a viral infection, patients with migraine and brain injury are more likely to have it than those without migraine (28). Different areas of the brain are activated depending on the various migraine symptoms, especially the trigeminovascular system, brain stem, hypothalamus, and cerebral cortex (27). SARS-Cov-2 may directly or indirectly affect these regions in the brain of migraineurs and may be responsible for migraine-like symptoms in association with COVID-19.

We observed a frequency of headache triggered by coughing of 16%, which is much higher than that observed in the general population (1%) (29). Most of our patients were aged over 50 years and were male. As cough headache is more frequent in males and those aged over 40 years, this may have contributed to the high frequency (29), although this does not fully explain the situation. We cannot rule out the fact that these patients may have had some degree of intracranial hypertension.

The frequency of hyposmia/anosmia and hypogeusia/ageusia observed in the present study is in line with that reported in the literature (12,30). Most patients with ageusia also had anosmia. The change in taste may be partly explained by the change in smell, since taste is influenced by the perception of odors.

Patients with these complaints presented a significantly higher frequency of headache and these symptoms had a close temporal relationship, mostly occurring at the beginning of the symptomatic phase of COVID-19. Another study also found a higher frequency of headaches amongst those who had anosmia (20). Most neurological symptoms are likely to occur in the early stages of the disease with a median of 1–2 days, as cited in a recent review (18).

The mechanisms of injury to the nervous system by SARS-Cov-2 are not yet fully understood. Direct injury of the peripheral and central nervous systems by viral infection via the neuronal pathway, notably the olfactory bulbs, and possibly also by the hematogenous pathway, seem to be responsible for the earliest neurological symptoms. Inflammatory responses, with the systemic and nervous system cytokine storm, seem to be responsible for the most severe forms of COVID-19 (2,18,31). As anosmia and ageusia are early symptoms, the association with headache in our patients may indicate that the virus itself must participate in the mechanism of developing pain and associated symptoms.

Patients with headache, hyposmia/anosmia and hypogeusia/ageusia reported significantly more phonophobia than those who presented no olfactory and gustatory dysfunction. This is the first time that an auditory symptom has been associated with other neurological symptoms previously described in COVID-19.

Phonophobia may be defined as the negative emotional reaction of hyperacusis (32). This symptom has also been reported in other infectious neuropathies, such as in the intracranial involvement of the facial nerve by reactivation of the herpes zoster virus in the geniculate ganglion (in these cases associated with dysgeusia), or in Lyme disease (33,34). SARS-Cov-2 has already been isolated from cerebrospinal fluid and other coronaviruses have been isolated in several regions of the central nervous system, such as the hippocampus and brainstem, suggesting a neurotropism of these viruses (2,35). Phonophobia observed in our patients raises the possibility that SARS-Cov-2 affects central regions involved in auditory processing.

This article has some limitations. We performed no olfactory and taste testing to objectively prove anosmia and hyposmia. As we did not calculate the sample size, we cannot rule out the possibility that the study could not identify small differences between the groups. The patients were seen in a single moment, while presenting with respiratory symptoms. Thus, the characteristics of headache may be changeable during the disease course, and the frequency of headache, anosmia, and ageusia may have been underestimated since there is a possibility that the patient continued to present these symptoms during the course of the disease. Nonetheless, the frequency of headache presented was higher than most studies. Most patients presented symptoms for more than 15 days and were already at the end of the symptomatic period when they were assessed.

Neuroimaging, cerebrospinal fluid tests or ophthalmoscopy were not performed to rule out other causes of secondary headaches such as meningitis (36), encephalitis, and cerebrovascular diseases (1), which may be complications of COVID-19. However, these patients had no meningeal or focal signs, confusional states, or impaired level of consciousness, which makes these complications less likely.

This study was conducted in a single center and involved only inpatients. This decreases the ability to generalize the study, and therefore extrapolating the results to patients with mild forms of the disease, who do not require hospitalization, should be undertaken with caution. However, a study that assessed symptoms of patients with mild forms of the disease, who did not need to be hospitalized, reported similar results to ours (headache: 70%; anosmia: 28% and ageusia: 28%) (37).

In conclusion, there was a high frequency of patients with COVID-19 presenting with headaches, generally very intense, diffuse and with a migraine phenotype. Those patients with anosmia and hypogeusia reported more headaches, which occurred in close temporal relationship with these symptoms and demonstrated more associated phonophobia. Headache associated with COVID-19 presented a migraine phenotype more frequently in those experiencing previous migraine.

## Clinical implications

- COVID-19 headaches were very frequent, generally began at the onset of symptoms, were bilateral, of moderate or severe intensity, throbbing, and with a migraine phenotype.
- Fifteen percent of the patients presented a continuous headache, which was generally moderate to severe, and lasted for at least 15 days.
- Sixteen percent of the patients presented headaches triggered by coughing.
- Those patients with anosmia and ageusia reported more headaches, which occurred in close temporal relationship with these symptoms and demonstrated more associated phonophobia.
- In patients who have migraines and headaches associated with COVID-19, this headache most frequently has a migraine phenotype.
